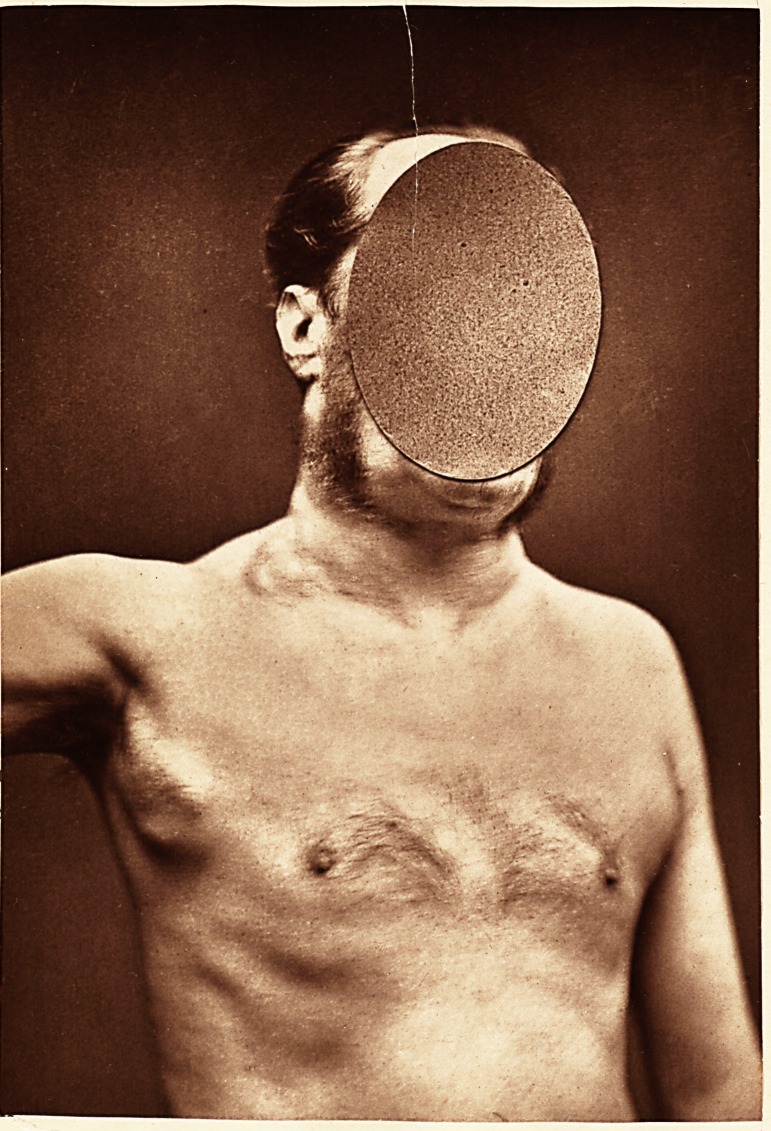# Enlargement of the Spleen

**Published:** 1885-09

**Authors:** E. Long Fox

**Affiliations:** Consulting Physician to the Bristol Royal Infirmary


THE BRISTOL

Medico-Chirurgical Journal.

SEPTEMBER, 1885.

### ENLARGEMENT OF THE SPLEEN.

BY

E. Long Fox, M.D., F.R.C.P.,

*Consulting Physician to the Bristol Royal Infirmary.*

It is no part of the present paper to deal with all the functions of the Spleen. That it may be removed without materially causing ill effects to the animal, is no proof that it is an useless gland. Such a fact is only an illustration of Nature's prevision, by which some other structure is arranged to supply the lapsed duties of a lost or injured organ. Let it be said, briefly, that although the medulla of the bones is the chief seat for the formation of the red corpuscles of the blood, yet this duty falls also, in smaller measure, to the share of the spleen; that the spleen is largely concerned in the breaking up of the red corpuscles, in which process hæmoglobulin is set free; that one of the derivatives of this free hæmoglobulin is hæmatoidine, which probably, under the name of bilirubin, supplies bile pigment; that the spleen's pulp, besides containing a special proteid associated with iron, is rich in several peculiar carbonaceous pigments; that although the formation of the white cells of the blood seems to be mainly the duty of the lymphatic glands proper, yet the spleen shares in this function.

It is not proven that any of these functions of the spleen go on actively when the organ is of normal size, and when the circulation in it simply follows the ordinary course of the vessels. It seems necessary for full functional activity that the meshes of the organ should be filled with blood from the vessels that open into its network; but this normal enlargement of the spleen occurs, probably, several times a day, and functional activity, therefore, is in no danger of falling into abeyance. Such a diurnal erethism seems to be active rather than passive, and may be induced by the irritation of vasodilator fibres in the solar plexus, or of fibres that have their origin in the medulla oblongata. Few, if any, of the abnormal enlargements of the spleen are due to an active erethism. An enlarged spleen may mean one in which there is simply great congestion of the organ; or, on the other hand, one in which there is much interstitial thickening. It may be an open question whether the latter form ever occurs without having been preceded by the former.

It will be seen that in almost all cases splenic dilatation is due to a paresis of vasoconstrictor nerves. Whether this be so when the spleen is doing little more than acting as a diverticulun, in cases of hepatic incompetence, may be doubtful. The enlargement is at first, at least, merely mechanical; but even here the secondary effects of vascular pressure will induce a vasomotor paresis. In taking cases almost haphazard from *post-mortem* records, personal observation would simply verify that of the profession at large. Thus, a spleen of large size was found coincident with hydatids of the liver; with a liver indurated from amyloid disease; with cirrhosis of the liver; with disease of heart, and liver of stony hardness as its consequence; with hepatic anæmia in general tuberculosis, in which the spleen, though not tuberculous, was especially large and firm; with fatty liver coincident with pleurisy and pericarditis; with the liver fatty and very small, in general tuberculosis, when the spleen weighed $1\frac{1}{4}$ lb., but was of soft consistence. These would be only a few instances, out of many, in which the size of the spleen has depended upon the incompetence of the hepatic circulation, if not of the hepatic function.

In tuberculosis, whether the spleen is itself the seat of tubercle or no, the bacilli groups, or the irritation of the degeneration of tubercle, may form the starting-point of reflex action, in which the solar plexus is the centre of the arc, and the splenic fibres the exodic nerves. In some cases of tuberculosis, I have found the spleen three times its usual size; in tuberculous ulceration of the intestines, with the liver healthy, the spleen was large, dark, and soft; in caseation of some of the vertebræ and of the kidneys, the spleen was as large as in severe cases of ague. In many cases this enlargement is associated with a profuse studding of the organ with miliary tubercle; but the size of the spleen seems in no way to be due to the presence of tubercle in its parenchyma. It is not common to find breaking-down of tubercle in the spleen; but one such case is recorded in an early note-book, where the spleen was the seat of very numerous minute tuberculous abscesses, more or less encysted. This specimen apparently resembled some described by Dr. Moxon, as to the explanation of which he throws out the suggestion that the little abscesses might have had an hydatid origin.

It is probably only in cases of tuberculosis, in which this reflex enlargement of the spleen occurs, that Dr. Gueneau de Massy's dictum holds good, that pigmentation in the face in phthisis is a sign of tuberculous ulceration of the intestines. This ulceration is frequently met with without any pigmentation of the face. The coincidence of the lesion with the physical sign is by way of irritation from the ulcer carried to the solar plexus, and reflected down upon the vasomotors of the spleen, causing dilatation of vessels, and hæmorrhagic distension of its meshes; and thus is brought about a necessarily preliminary condition for the setting free of pigment.

It may be a doubtful point whether, in fever, the microbe, the condition of blood engendered by it, or, in the case of typhoid, the intestinal ulceration, is the starting-point of a similar irritation of the solar plexus and semilunar ganglion, that results in dilatation of the splenic vessels and enlargement of the spleen. I have seen the spleen a foot long in maculated typhus. In a case of septicæmia following the bite of a rat, and in which the symptoms for some time closely simulated typhoid, the spleen could be marked out by palpation, and was greatly enlarged. In neither of these two latter morbid conditions should we be likely to have any intestinal ulceration. The probability, therefore, is, that in typhoid itself it is not the ulceration that is the starting-point, but rather the condition of the blood itself. It is, of course, a matter of daily experience to find this enlargement of the spleen in typhoid patients in life: it would be an exception not to find it *post-mortem* in those cases that die in the third or fourth week. In some few cases the liver may be pale and bloodless, but this is not the rule; and note-book records speak of the spleen in typhoid weighing 15 oz., 1 lb., 1 lb. 2 oz.—the organ being long and large, mottled, some parts being darker than others—1 lb. 4 oz., $3\frac{1}{2}$ lb., &c. But in fever the spleen will sometimes far exceed these weights. In fever, especially typhoid and relapsing fever, it is acute swelling of the spleen that is usually met with. The organ is soft, the pulp-cells become swollen and granular, and, Hoffman states, contain red corpuscles in their protoplasm. Although, in these febrile diseases, there are seldom any isolated patches of pigmentation of the cutaneous surface, yet there is a peculiar discolouration of the skin, quite different from mere venosity, and that gives the idea of the subcutaneous vessels being filled with a blood highly charged with pigment.

The spleen, too, is sometimes found enlarged when some portion of the body is the seat of malignant disease. In some cases it may be enlarged, and be itself infiltrated or spotted with cancer. But the presence of this morbid growth in its parenchyma seems to have little to do with its enlargement. Occasionally this condition has seemed to depend on the incompetence of the liver, this latter organ being spoiled by cancerous substance in it. In one case the spleen was found very large, but free from malignant disease, whilst the liver was much affected with it, and the lungs were very unusually pigmented. In another, the liver was highly cancerous; in another, with a spleen large and firm, but otherwise healthy, the liver, uterus, and mesenteric glands were the seat of cancer. Here, again, the condition of blood, consequent on the presence of malignant disease, affects either the solar plexus or more directly the sympathetic nerves of the spleen; and it is this frequent association of cancer or sarcoma with enlarged spleen that affords some explanation, not only of the peculiar cachectic sallowness, so useful for diagnosis, but of much more marked and isolated pigmentation, or of the rapid increase of congenital pigmental markings, that may be frequently recognised in malignant disease, more especially in melanotic sarcoma.

In a short paper it would be difficult to discuss all the varieties of splenic enlargement. Much might be said of primary splenitis, due mainly to hæmorrhagic infarctions, of which suppuration may be the ultimate consequence; or of secondary splenitis, depending on pyæmic infarctions, which proceed very rapidly to suppuration. The size of the spleen under either of these conditions is extremely variable, but it may occasionally form a tumour that occupies the whole of the left side of the abdominal cavity.

In rachitis, the enlargement of the spleen may be considerable. In a child of two years old, this organ was found nine inches long and four in its greatest breadth. In this condition the spleen will sometimes occupy a large part of the abdominal cavity, and it is then, pathologically, closely allied to the spleen of chronic ague, in which the trabecular stroma and the lymphoid tissue increase pretty equally.

In syphilis, a similar condition may obtain, whether the organ itself is the seat of syphilitic deposit or no. But this enlargement of the spleen is not very common; when it is met with, it is in the tertiary stage of syphilis. In one case I have noted a large organ with thickened capsule, and, on section, several yellowish-white deposits. But Wilks and Moxon state that, under these circumstances, hypertrophic enlargement of the spleen is often met with, and this in bodies quite free from amyloid disease. In eight such syphilitic subjects, the average weight of the spleen was 19 oz., whilst there was neither cirrhosis of liver nor lardaceous disease present in them.

In lardaceous disease, the size of the spleen may exceed the normal; it may take the form of sago spleen, the malpighian bodies being filled with this albuminoid material; or the lardaceous degeneration may occur in a diffused form throughout the whole of the spleen, this organ being then large, anæmic, and presenting, on section, the appearance of raw bacon.

Infarctions of the spleen are not confined to splenitis; they are found in several other enlargements of spleen, and are not uncommon in leucocythæmia; they are sometimes embolic, sometimes thrombotic. Dr. Goodhart's explanation of the course of the lesion is as follows: "The ultimate ramifications of the splenic artery are terminal; that is to say, they do not anastomose with each other. If one of them becomes blocked by an embolus, the circulation beyond is entirely arrested, and the blood-vessels, thus deprived of their function, speedily fall into a state of impaired nutrition. After a short time, the vein which drains the affected area, still receiving blood from the collateral branches, allows of a retrograde current, at first, according to Cohnheim, partial but continuous, then rhythmical. Thus the blood, finding its way back into the empty and feeble vessels, becomes extravasated, and an infarction is the result. It is but seldom that any other termination than gradual resolution occurs; but should the plugging be composed of septic or foul material, an abscess may result."

In acute ague, the spleen is large and generally soft: it is not in this stage that the trabeculæ become affected.

In chronic ague, there is often thickening of the capsule, a considerable increase in the fibrous trabeculæ, with thickening of the arterial and venous walls, with proportional augmentation of the lymphoid tissue. The enlargement of the organ may be very great, and is frequently associated with the formation of a large amount of pigment. Whether this is, as Wedl believes, a parenchymatous inflammation or no, and whether its starting point is a direct action of a vegetable organism on the splenic ganglia and tissues, or a consequence of such action of a vegetable organism on the semilunar ganglia or the sympathetic of the spleen, the difficulties in the comprehension of ague remain unanswered. The splenic enlargement may be reduced by treatment; the patient may live for many years outside the influence of malarial poisoning; and yet, whenever such a patient gets out of health in any way, the intermittent symptoms will recur. Do the organisms that induce the symptoms of ague exist everywhere in minute amount, and in healthy districts only exercise their sway upon those persons whose nervous system has been weakened by previous attacks, and on them only when nervous energy is at the lowest ebb? Or do some of these organisms always remain somewhere (perhaps in the spleen) in a patient who has once yielded to the effects of malarial poisoning, ready to increase and multiply at any moment of the depression of nervous force? Either theory is beset with difficulties. The latter theory is held by Dr. Fyffe. He also referred, in his presidential address, to the enormous size attained by the spleen sometimes in this chronic form of ague; and he mentioned a specimen in the Pathological Museum of the Army Medical Department, where the spleen of a boy, aged 14, weighed 10 lb. 15 oz., and measured 14 in.

by $7\frac{3}{4}$ in. The pigmentation of the cutaneous surface and of internal viscera, in chronic ague, is notorious.

The other main examples of enlargement of the spleen associated with distinct abnormal increase of growth of some of the tissues of the organ, are seen in leucocythæmia and in lymph-adenoma. This last morbid state—the adénie of Trousseau—may assume a specially lymphatic or a specially splenic form, but the two are more frequently combined. The hypertrophy of the spleen is a gradual chronic process. It is not extreme. The spleen seldom attains even the ordinary size of the organ in chronic malarial poisoning. It differs also from this, in that its abnormality consists in the increase of its lymphoid elements, and that there is but seldom any marked augmentation of the trabecular substance.

In a case that was under observation for more than four years (and of which a photograph of the external appearance of some of the lymphoid glands is appended), the *post-mortem* record was as follows: Body much emaciated; enlarged glands in the cervical, axillary, and inguinal regions; one prominent gland on the left instep; bronchial glands enlarged, about the size of a haricot bean and upwards. Heart: several old bands of adhesion between the apex of the heart and the pericardium, occupying about a square inch of space; heart pale, flabby, and loaded with fat; mitral and semilunar valves healthy; tricuspid valve had an atheromatous patch, about the size of a split-pea, on the middle cusp. Remains of old pleurisy on the left side, of recent pleurisy on the right. Some emphysema of the upper portion of right lung; the lower lobe hepatised. Liver: some whitish patches, quite superficial, about the size of a shilling, along the anterior margins; otherwise normal. Kidneys the right, of normal size; the capsule stripped off easily: three large cysts full of fluid, the largest as big as a walnut; very little normal renal structure left; left, of normal size; a few cysts, smaller than those of the right kidney. Spleen: about three times the normal size, rather pale; otherwise, nothing abnormal apparent to the naked eye, except an infarctus of some size in its centre. The enlargement was found to be due to a great increase of the lymphoid elements. The splenic rete was nowhere hyperplasic. The mesenteric and other abdominal glands were much enlarged, and of various sizes. In none of the glands examined—axillary, bronchial, and mesenteric—was there any hyperplasia of the connective tissue, only a great increase in the cell elements. The bronchial glands were extremely pigmented A feature in the case was the very marked diminution in the size of the external glands and of the spleen during the last two weeks of life.

In many cases, however, the spleen does not attain to anything like this size; but even in the smaller spleen, associated with Hodgkin's disease, the lymphoid tissue around and in the immediate neighbourhood of the arteries is moderately increased, and, taking on a somewhat nodulated appearance, gives rise to what has been termed the hard-bake spleen.

I have only once seen an autopsy of the very acute form of Hodgkin's disease. Death ensued from mechanical causes. Not only were the axillary glands exceedingly enlarged, but the bronchial and mediastinal glands rapidly attained such a size as to compress the inferior vena cava, whilst at the same time the return of the blood from the head was materially interfered with by the extreme enlargement of the cervical glands. In this case no abnormality of the spleen could be detected by palpation. The cavity of the thorax was alone allowed to be examined.

In leucocythæmia the lymphatic glands are frequently enlarged, though their structure varies but little from that of ordinary adenoid tissue in health. The marrow of the bones presents a semi-purulent appearance, from hyperplasia of its lymphoid elements, and the spleen is usually exceedingly enlarged, though still retaining, more or less, the shape of the normal organ. It is common to find traces of adhesions to the surrounding organs, and sometimes some thickening of its capsule. The trabecular tissue is hyperplasic; there are frequently to be found infarctions, more or less considerable in size, from plugging of the splenic vessels with white cells, as in other organs and tissues of the body in this affection. In the early stage there may be enlargement, with a normal consistence, the organ containing a good deal of blood. As the disease becomes more chronic, induration occurs, with greater hyperplasia of the trabeculæ. The enlargement is due to the presence of great numbers of lymphoid cells in the meshes of the retiform tissue. In this form of splenic enlargement there seems to be but seldom any abnormal pigmentation. May not this be explained by the fact that the normal functions of the spleen are prevented by the presence of the large numbers of lymphoid cells?

The theory has been advanced of a connection between the leucocythæmic spleen and exposure to malarial poison. The absence of pigmentation in leucocythæmia militates, to some extent, against this view; but, as a matter of observation, a considerable proportion of patients suffering from leucocythæmia have either been ague-stricken or, at least, have lived in malarial regions. I have met with one rather rapidly fatal case, in which, four years previously, one of the mammæ had been removed for scirrhus, and, at the time when a return of malignant disease might have been expected, leucocythæmia supervened.

In a case lately seen, where the patient had been ill for a year, the heart was dilated, there was a hæmic bruit, the liver was enlarged, the spleen occupied most of the left half of the abdomen, the proportion of white cells in the blood was much increased, and there was great debility and a very cachectic look.

In another case, with similar symptoms, the spleen, which had been very large, became reduced in size, whilst the increased proportion of the liver persisted. The case was lost sight of before death; but a similar thing had been noticed some years ago in the Bristol Royal Infirmary, and it was found, after death, that the enlarged spleen had become closely united to the walls of the stomach, that ulceration of the latter had taken place, that the grumous softened spleen (not by any means a common condition of leucocythæmia) had, to a great extent, emptied itself, as regards its exaggerated lymphoid elements, into the stomach, and that this softened material had passed per rectum.

In another case—that of a clergyman, in whom the disease lasted little more than a year—the spleen intruded quite into the pelvis, and the proportion of white cells in the blood was very large. Although there could be detected no inflammatory complications, the evening temperature during the last two months of life varied from 2° to 5° Fahr. above the normal standard.

Both during life and in the dead-house, the connection between leucocythæmia and Hodgkin's disease seems to be close; and yet, in the latter, any increased proportion of white cells in the blood is of the faintest kind; whilst the spleen is far more capable of normal function, and pigmentation (sometimes of the skin, more frequently of some of the internal organs) is more commonly found. Fatty degeneration of the heart is more usual in leucocythæmia than in adénie, and also minute extravasations of blood in the brain, the meninges, and elsewhere.

It is a question whether it is right to speak of the melanæmic enlarged spleen as an entity, separate and distinct from ague. It is met with most usually in severe fevers of a remittent type; but, on the other hand, in melanæmia, not only are the white cells of the blood found in an abnormal proportion, because of the diminution of the red cells, but there is an absolute increase in their number. The spleen, in this disease, is enlarged and indurated, with hyperplasia of the rete; it is crowded with pigment in flakes, in free granules, and in granules contained in the white cells. This pigment is found in most of the other organs in the body, notably in the liver, the lungs, the brain, and the marrow of the bones; but the spleen is not only the most pigmented of any organ, the pigment infiltrating the whole viscus, affecting especially the immediate neighbourhood of the arteries and veins; but it is the only organ decidedly diseased.

It is, at least, plausible to believe that most of the pigment deposited in other organs issues from the disordered spleen, though Planer, Frerichs, and others deny this. The weight of authority, however, is in favour of the splenic theory. Colin considers that the destruction of red cells, and the formation of pigment, in the spleen is caused by a violent congestion of the blood in this organ, but that a similar process goes on in other organs where pigment is found. It seems more in accordance with natural law that the same process should be set in action in all pigmentation. There is no special congestion of the skin in Addison's disease before the bronzing commences, although some stasis of circulation is a necessary element in the deposit of pigment anywhere.

Pathology and pathological anatomy seem to teach that, whatever be the abnormalities of circulation or of vascular innervation that determine the topical deposit of pigment, the primary causation of its abnormal development lies in a direct or reflex irritation of the solar plexus, transmitted to the sympathetic plexus of the spleen, paralysing or rendering paretic the vasoconstrictors of this organ, determining congestion, and in some instances, as a result of long-continued congestion, a hyperplasia of the stroma and of the splenic pulp, leading to a great destruction of red globules, and a production of a larger amount of pigment than is required for the ordinary colouring of the secretions, especially of the hepatic.

In this relationship the germs of fever, of septicæmia, &c., directly or indirectly affect the spleen; congestion of this organ, resulting from the mechanical effects of incompetent liver, diseased heart, impeded portal circulation, gives rise to a similar functionally-abnormal phenomenon. The bacillus of tubercle, the microbe of syphilis, or the state of blood engendered by them, the cachexia of sarcoma, may, in certain cases, lead to a similar enlargement of the spleen. A vegetable organism may be the starting-point of irritation in ague and in melanæmia; whilst in adénie and in leucocythæmia, the existing cause has not yet been recognised. Irritation of the solar plexus, and consequent paretic dilatation of the splenic vessels, seems to be the factor usually found in the pathological anatomy of pigmentation, and it affords a further example of the economy in natural processes.

## Figures and Tables

**Figure f1:**